# Porous Silicon-Based Biosensors: Towards Real-Time Optical Detection of Target Bacteria in the Food Industry

**DOI:** 10.1038/srep38099

**Published:** 2016-11-30

**Authors:** Naama Massad-Ivanir, Giorgi Shtenberg, Nitzan Raz, Christel Gazenbeek, Dries Budding, Martine P. Bos, Ester Segal

**Affiliations:** 1 Department of Biotechnology and Food Engineering, Technion – Israel Institute of Technology, Haifa 3200003, Israel; 2 The Interdepartmental Program of Biotechnology, Technion – Israel Institute of Technology, Haifa 3200003, Israel; 3 Microbiome Ltd, Amsterdam, the Netherlands; 4 Department of Medical Microbiology, VU medical Center, Amsterdam, the Netherlands; 5 The Russell Berrie Nanotechnology Institute, Technion – Israel Institute of Technology, Haifa 3200003, Israel.

## Abstract

Rapid detection of target bacteria is crucial to provide a safe food supply and to prevent foodborne diseases. Herein, we present an optical biosensor for identification and quantification of *Escherichia coli (E. coli*, used as a model indicator bacteria species) in complex food industry process water. The biosensor is based on a nanostructured, oxidized porous silicon (PSi) thin film which is functionalized with specific antibodies against *E. coli*. The biosensors were exposed to water samples collected directly from process lines of fresh-cut produce and their reflectivity spectra were collected in real time. Process water were characterized by complex natural micro-flora (microbial load of >10^7^ cell/mL), in addition to soil particles and plant cell debris. We show that process water spiked with culture-grown *E. coli*, induces robust and predictable changes in the thin-film optical interference spectrum of the biosensor. The latter is ascribed to highly specific capture of the target cells onto the biosensor surface, as confirmed by real-time polymerase chain reaction (PCR). The biosensors were capable of selectively identifying and quantifying the target cells, while the target cell concentration is orders of magnitude lower than that of other bacterial species, without any pre-enrichment or prior processing steps.

Recent reports estimate that about one-third of the food produced globally for human consumption is lost or wasted^1^,^2^. These losses occur at all stages of the food value chain and across all types of food, resulting in wastage of natural resources such as water, energy, and soil^3^. These alarming figures combined with recent estimations that the water requirements to meet food demand in 2050 may triple the current annual consumption^4^ reveal the urgent need to reduce the water footprint in both horticulture and the food industry^3^. Thus, increasing the reuse of water during food production and processing is an urgent need in the global effort to reduce food waste and improve sustainability^5^,^6^. A significant challenge for the development and operation of water reuse schemes is to ensure water quality and safety via appropriate monitoring techniques^7^,^8^,^9^. In particular, because microbiological quality assessment of food and water continues to rely on traditional culture-based techniques (which require at least 24 h to obtain results), real-time or near real-time evaluation of reused water safety remains a challenge^10^,^11^. Although advanced techniques in microbiology, e.g., biochemical kits, ELISA (Enzyme-Linked Immunosorbent Assay) and PCR (Polymerase Chain Reaction), have shortened assay time, they still lack the ability to detect microorganisms in real time or on-site^12^,^13^,^14^,^15^. Therefore, there is an unmet need for rapid, direct (no pre-cultivation enrichment), reliable, and portable methods to evaluate real-time quality and safety of water and food^16^. Biosensors offer significant advantages in microbial analysis of water and food samples, as they can include fast or real-time detection, portability, and multi-pathogen detection^11^,^17^,^18^,^19^,^20^,^21^. Such systems can potentially be deployed at food processing plants and food safety laboratories to allow for rapid detection of foodborne microorganisms and enhance food safety^22^,^23^.

Our work focuses on the development and application of a novel biosensing platform for rapid detection and identification of microbial contaminations in complex food industry process water (Fig. 1a). The optical, label-free biosensing platform is based on a nanostructured, oxidized porous silicon (PSi) designed to directly capture the target bacteria cells onto its surface with no prior sample processing^24^,^25^,^26^,^27^. PSi-based Fabry-Pérot thin films are functionalized with specific antibodies to act as the active component of the biosensor (Fig. 1b). White light is focused onto the biosensor and the optical data is collected throughout the experiment. The obtained reflectivity spectra are comprised of a series of Fabry-Pérot interference fringes^28^, resulting from reflections at the top and bottom interfaces of the porous thin film (Fig. 1c, upper panel). The collected spectra are analyzed by applying a fast Fourier transform (FFT), which results in a single peak with a characteristic effective optical thickness (EOT) and intensity (Fig. 1c, lower panel)^29^,^30^. The position of the peak along the *x*-axis of the FFT spectrum corresponds to the EOT value, which equals 2*nL* (where *n* is the effective refractive index of the porous film and *L* is its physical thickness)^28^. Introduction of the target bacteria to the biosensors results in their capture onto the biosensor surface. These specific binding events induce predictable changes in the thin-film optical interference spectrum of the biosensor, i.e., a decrease in the amplitude (intensity) of the reflected light (Fig. 1c). Our recent work has demonstrated the potential of these PSi-based biosensors to detect *E. coli* bacteria in laboratory suspensions at relatively low bacterial concentrations (in the range of 10^3^–10^5^ cells/mL) within minutes^25^,^26^,^31^.

In this new work, the biosensors were redesigned in terms of their surface chemistry and their ability to detect target bacteria within “real” process water (derived directly from the process line of fresh-cut produce industry) is studied (Fig. 1c). The bacterial profiles of the process water were determined by both conventional culturing technique in addition to a new polymerase chain reaction (PCR) based technology, IS-Pro^32^. We demonstrate rapid detection of *E. coli* (used as a model indicator bacteria) via a “direct cell capture” approach onto these biosensors. *E. coli* was used in this work as the target microorganism as it is considered as indicator bacteria for fecal contaminations^33^,^34^,^35^ and recognized as an important foodborne pathogen associated with fresh produce with very low infectious dose^36^. To achieve this goal, oxidized PSi films (PSiO_2_) were fabricated and biofunctionalized with specific antibodies against *E. coli*. Changes in the intensity of the reflectivity spectrum of the biosensor were monitored in real time upon exposure to process water containing *E. coli* bacteria (in addition to its high natural microbial load). Correlation between the measured light intensity and specific capture of the bacteria onto the biosensor allows for rapid detection and quantification of bacterial contaminations. The capture of the target *E. coli* cells onto the biosensor was confirmed and quantified by real-time PCR. This work sets the foundation for implementing a one-step and rapid biosensing platform in the food industry.

## Results

### Process water characterization

**Figure 1:**
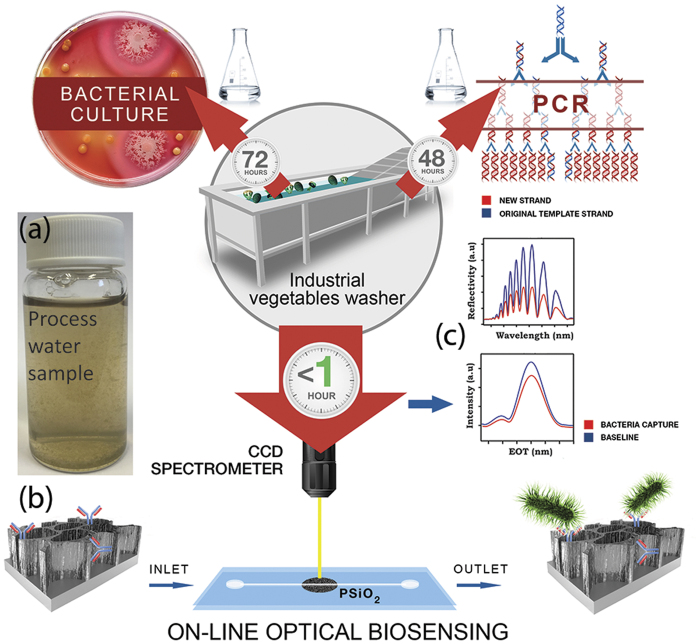
(**a**) Water samples from a Dutch fresh-cut produce company were evaluated for *E. coli* presence by three different methodologies: culturing techniques (upper left), PCR-based methods (upper right), and label-free optical biosensors (bottom). Note that the time indicated for each method refers to the total assay time. (**b**) Specific capture probes (antibodies) immobilized onto the PSiO_2_ surface function as the active component of the biosensor. After exposure of the biosensor to process water spiked with the target bacteria, the bacteria cells were directly captured onto the antibody-modified PSiO_2_ surface. (**c**) Light reflected from the porous nanostructure provides the monitored optical signal. Changes in the light intensity are correlated to specific immobilization of the bacteria onto the surface. Upper panel: reflectivity spectra of a typical Fabry-Pérot PSiO_2_ nanostructure before (blue) and after (red) bacteria capture. Lower panel: applying a fast Fourier transform (FFT) of the raw reflectivity spectrum results in a single peak whose magnitude is monitored.

Water samples from a fresh produce processing company were sampled from different washing lines and characterized by three different methodologies: culturing techniques, PCR methods, and by our label-free, optical biosensing platform (see Fig. 1).

**Figure 2:**
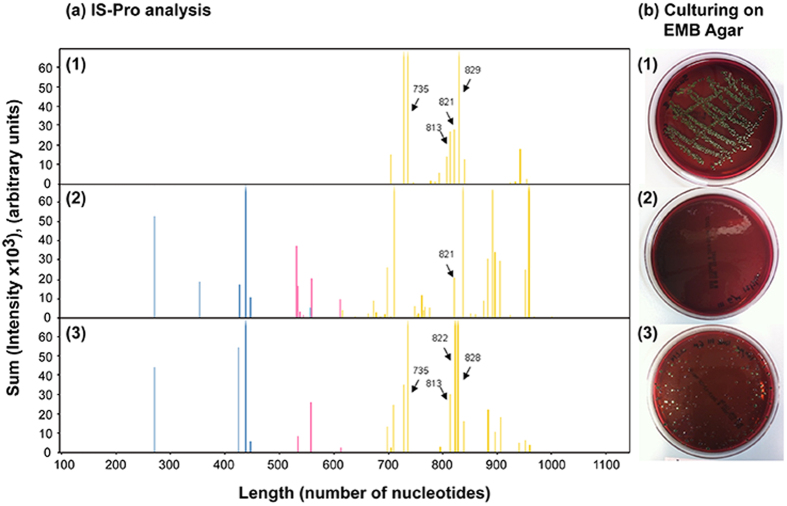
(**a**) IS-Pro bacterial profiles and (**b**) the corresponding *E. coli*-specific medium culture (EMB agar) of (1) pure *E. coli* K-12 culture; (2) water samples before spiking with *E. coli* K-12; (3) water samples after spiking with 10^5^ cells/mL *E. coli* K-12. Peak length, expressed in nucleotides, corresponds to IS-fragment length. Peak height, expressed as intensity, reflects quantity of fragments. The blue peaks represent *Firmicutes*, pink peaks represent *Bacteroidetes,* and yellow peaks represent *Proteobacteria*. The *E. coli*-specific peaks are indicated by arrows and amplicon length.

**Figure 3:**
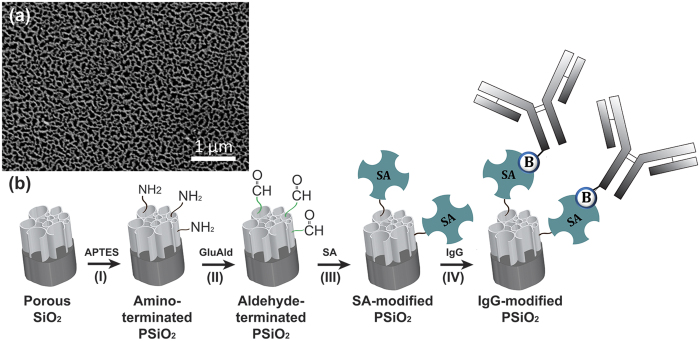
(**a**) A top-view high-resolution scanning electron microscope (HRSEM) image of a typical PSiO_2_ film demonstrating the porous nanostructure morphology with typical pores in the range of 60–100 nm. (**b**) Schematic illustration of the synthesis steps for the biofunctionalizion of PSiO_2_ with IgG. (I) PSiO_2_ was reacted with APTES and catalyzed by an organic base to create an amine-terminated surface. (II) The amine-terminated PSiO_2_ was reacted with one of the aldehyde groups of the cross-linker GluAld. (III) Grafting of SA onto the aldehyde-terminated surface. (IV) Biotinylated-IgG (*E. coli*) was conjugated via biotin-SA binding.

The bacterial load in the process water, as determined by culturing on plate count agar (PCA) medium, was approximately 5 × 10^7^ cells/mL. It is important to note that the actual number of live bacteria in the process water is probably much higher, as many bacteria species are considered as “unculturable” using current laboratory culturing techniques^37^. Bacterial population was characterized by using a new PCR-based profiling technique (IS-Pro)^32^ and the results are presented in Fig. 2a-2. In brief, the profiling is based on species-specific length polymorphisms of the interspace (IS) region (the IS region between 16 S and 23 S rRNA genes) and phylum-specific sequence polymorphisms of 16 S rRNA gene. Amplification of the IS region with fluorescently labeled phylum-specific primers yields peak profiles of the different bacteria species that the water contain (see Fig. 2a-2). The Is-Pro bacterial profile confirmed the presence of *Firmicutes, Bacteroidetes* and *Proteobacteria* in the water, while no *E. coli* was detected (in agreement with culturing results using *E. coli* specific medium, see Fig. 2b-2). For biosensing experiments, the process water were spiked with different concentrations of *E. coli* K-12 bacteria. The presence of *E. coli* in the spiked water was confirmed by both IS-Pro analysis and culturing (see Fig. 2a-3 and 2b-3).

### Rapid detection of *E. coli* in process water

#### Preparation of biosensors

Biosensors were prepared from PSiO_2_ Fabry-Pérot thin films. The porous nanostructure was formed by anodization of a p-type Si wafer at a constant current density of 385 mA/cm^2^ for 30 s, followed by thermal oxidization to render the surface into SiO_2_^26^. The morphology of the resulting porous nanostructure is presented in Fig. 3a. *E. coli* antibodies (IgG) were grafted onto the neat PSiO_2_ films using silanization chemistry and subsequent biotin-streptavidin (SA) coupling^38^. The detailed synthesis scheme is outlined in Fig. 3b. Briefly, biofunctionalization of the films was achieved by silanization with 3-aminopropyl(triethoxyl)silane (APTES) in order to form the amine-terminated PSiO_2_ (Fig. 3b-I) followed by exposure to glutaric dialdehyde (25 wt%) (GluAld) in order to form aldehyde-modified surface (Fig. 3b-II). Next, SA was attached to the aldehyde groups through its free amines, forming imine bonds^39^ (Fig. 3b-III). Lastly, due to their exceptional binding strength and specificity^40^, SA-biotin interactions were used to conjugate the biotinylated *E. coli* antibodies onto the nanostructure, (Fig. 3b-IV).

The binding of the *E. coli* antibodies (produced in rabbit) to the PSiO_2_ nanostructure was confirmed by exposing the biosensors to a fluorescently tagged anti-rabbit IgG followed by observation of the films under a fluorescence microscope. The fluorescence was quantified by image analysis (performed by Imaris Bitplane scientific software). Figure S1 (Supplementary Information) summarizes the results of these experiments, confirming the conjugation of the *E. coli* IgG to the SA-modified porous nanostructure and their activity. Control experiments using a fluorescently tagged anti-mouse IgG resulted in a negligible fluorescence signal indicative of the specificity of the IgG-modified PSiO_2_ biosensor, see Figure S1. Moreover, when the IgG conjugation step to the nanostructure was omitted, no fluorescence is detected, demonstrating the lack of unspecific binding between the anti-rabbit IgG and the SA-modified PSiO_2_ surface.

#### Biosensing experiments

**Figure 4:**
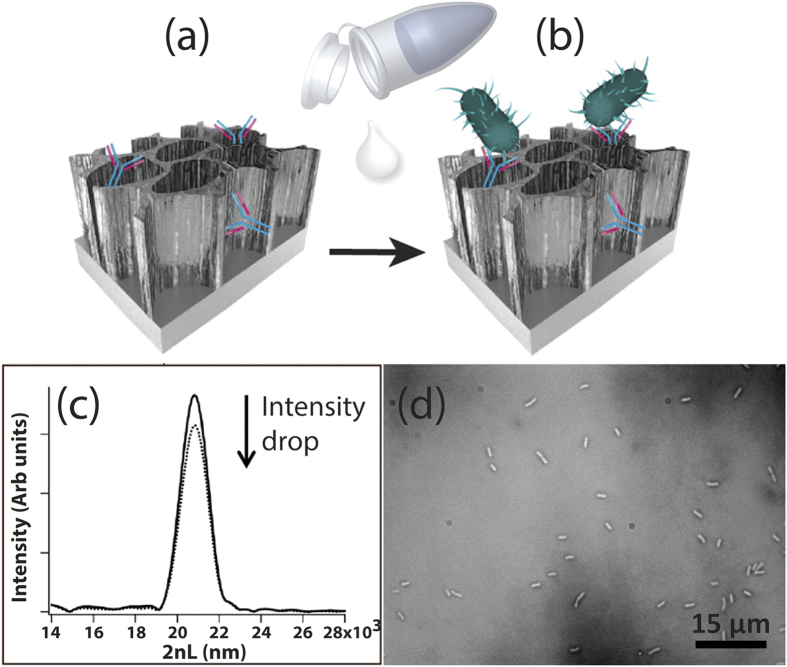
The biosensing concept. (**a**) Specific capture probes (antibodies) were immobilized onto the porous surface to provide the active component of the biosensor. (**b**) Next, the biosensor was exposed to the target bacteria in order to directly capture the bacteria cells onto the antibody-modified PSiO_2_ surface. (**c**) A drop in the intensity of the thin-film optical interference spectrum of the biosensor results from bacteria capture. (**d**) Microscopy tools (light microscope and HRSEM) and real-time PCR methods were used to confirm the presence of bacteria on the biosensor surface.

Our biosensors monitor changes in light reflected from the IgG-modified PSiO_2_ nanostructure, as shown in Fig. 4. Specific immobilization of *E. coli* cells onto the biosensor surface via antibody-antigen interactions induces a decrease in the amplitude (intensity) of the FFT peak of the porous film (Fig. 4c). These optical changes are correlated to cells captured onto the biosensor, clearly observed by optical microcopy studies (Fig. 4d)^24^,^25^,^26^,^27^.

**Figure 5:**
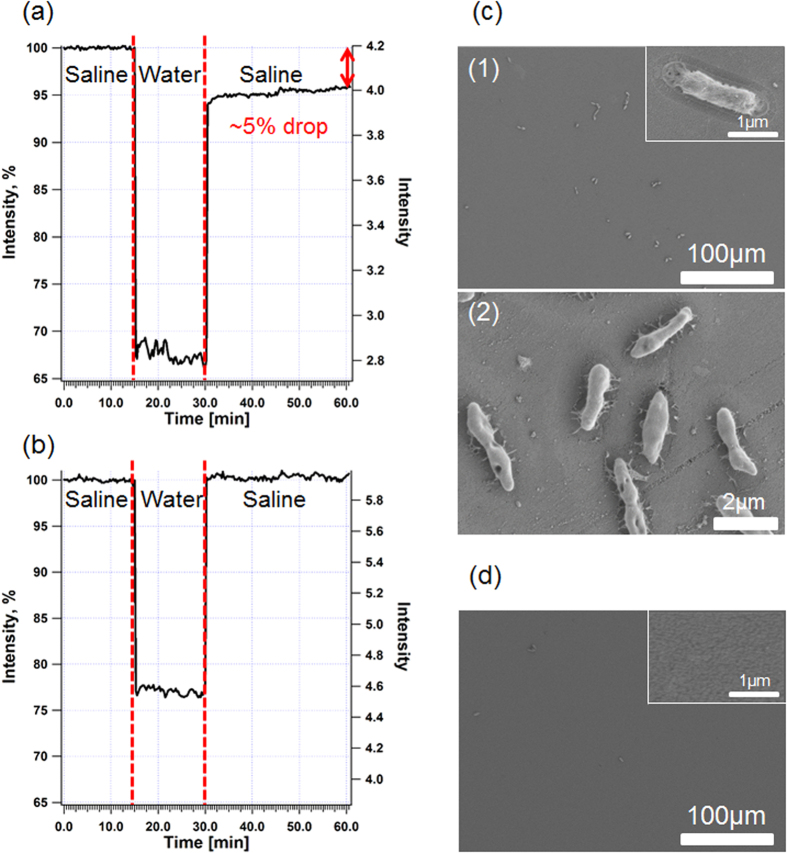
Representative biosensing experiments (left) and the corresponding HRSEM images (right) of the biosensors immediately after the experiments. (**a**) Spiked process water (10^4^ cell/mL *E. coli*). (**b**) Control - original process water (no *E. coli*). (**c**) The corresponding HRSEM images (in two different magnifications) of the biosensor after a biosensing experiment with spiked process water, demonstrating bacteria capture. The inset presents enlargement of a captured bacterium on the biosensor surface. (**d**) A corresponding HRSEM image of the biosensor after the control experiment (original process water, no *E. coli*) showing a negligible amount of cells. The inset presents enlargement of the biosensor surface.

The biosensors were incubated with process water spiked with *E. coli* K-12 bacteria (10^3^ to 10^5^ cells/mL) and the reflectivity spectra of the biosensors were collected using a CCD spectrometer and analyzed by applying a FFT. The incubation time was set to 15 min, after which the samples were washed with a buffer solution for 30 min. Figure 5 presents the results of a typical biosensing experiment (*E. coli* concentration of 10^4^ cells/mL) in terms of changes in the FFT peak intensity vs. time. First, saline was introduced into the flow cell to acquire a stable intensity baseline. Upon process water introduction, a rapid decrease in the intensity (~30%) can be observed (Fig. 5a) due to light scattering. The optical signal remained constant during incubation, but following a washing step designed for removal of unbound bacteria as well as dirt and plant cells debris from the biosensor, the intensity has increased significantly until attaining a new baseline corresponding to a net intensity decrease of 5%. This significant intensity change is attributed to binding of the target bacteria to the IgG-modified surface^25^,^26^,^27^. When the original process water (no *E. coli*) were introduced onto the biosensor, a similar trend of a rapid decrease in the intensity is observed followed by a plateau during incubation (Fig. 5b). However, upon removal of the water and rinsing, the intensity is observed to return to its original baseline value, suggesting that no capture events have occurred.

To validate the results of the biosensing experiments, the biosensors were studied immediately after the optical experiments by both light microscopy and high-resolution scanning electron microscopy (HRSEM). Figure 5c presents HRSEM images of the biosensor exposed to the process water spiked with *E. coli*. A large number of intact immobilized bacteria cells are observed throughout the biosensor surface (Fig. 5c), while no cells are seen on the biosensor incubated with the original process water (Fig. 5d). In order to confirm that the captured cells are indeed *E. coli* K-12 bacteria, the cells were recovered from the biosensors and analyzed by real-time PCR. The results are summarized in Table 1, revealing that the DNA extracted and isolated from the biosensors indeed belongs to *E. coli* K-12.

**Figure 6:**
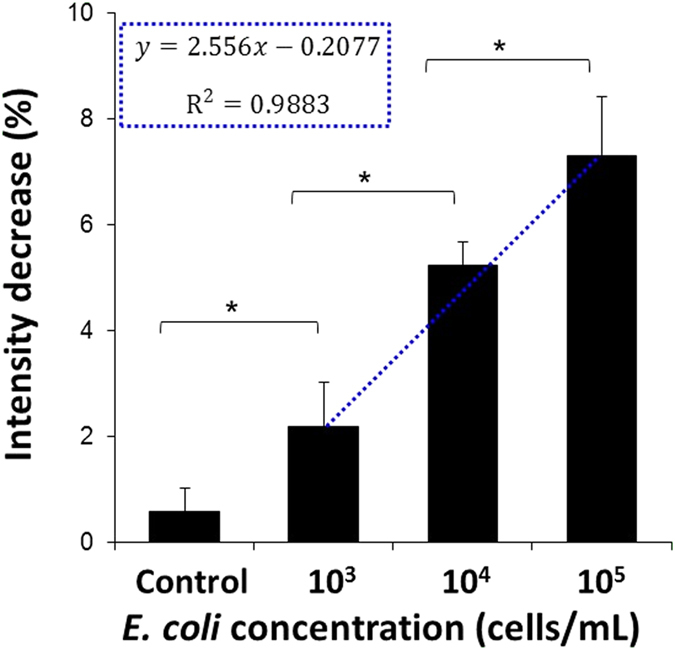
Averaged intensity changes of the biosensor upon introduction to process water spiked with different concentrations of *E. coli* bacteria (ranging from 10^3^ to 10^5^ cells/mL). For the control experiments, the biosensors were incubated with the original process water (no *E. coli*). The incubation time was set to 15 min, after which the samples were washed with a buffer solution for 30 min (n ≥ 3 for each concentration), *Significantly different (t-test, *p* < 0.05).

Figure 6 depicts the optical response in terms of intensity decrease when the biosensors were exposed to process water samples spiked with different concentrations of *E. coli* K-12 (ranging from 10^3^ to 10^5^ cells/mL). The biosensor response is found to be proportional to *E. coli* bacterial concentration and a linear correlation is demonstrated (R^2^ = 0.98). Importantly, significant statistical differences (*p* < 0.05) are found between the intensity signal values measured for process water containing different *E. coli* concentrations. In terms of the sensitivity of the biosensor, our experiments show a relatively low detection limit of 10^3^ cells/mL.

## Discussion

During the different stages of the food value chain over 40% of produced food is lost and wasted. This alarming statistic signals the need for a global effort and systemic approach to minimize losses throughout the food chain^3^. Moreover, in terms of global burden of foodborne diseases, according to the WHO (World health Organization)^41^, 600 million foodborne illnesses and 420,000 deaths were reported only in 2010. Therefore, developing advanced biosensing systems that can rapidly detect foodborne pathogens as early as during food processing and production is imperative for improving both food sustainability and safety^3^,^4^. This work focuses on the development and application of a new biosensing platform for rapid detection and identification of microbial contaminations in complex food industry process water. The water samples were collected directly from the process line of fresh-cut produce industry and thoroughly characterized in terms of their microbial profile by both conventional culturing technique and by IS-Pro analysis. This study presents a detailed comparison between biosensing results and gold-standard microbiological techniques (culturing and PCR) and a state-of-the-art microbiological method (IS-Pro). The total bacterial load of the process water as determined by culturing methods was approximately 5 × 10^7^ cells/mL. The IS-Pro bacterial profile of the process water confirms the presence of diverse bacteria phyla and species, such as *Firmicutes, Bacteroidetes* and *Proteobacteria*, see Fig. 2a-2. *E. coli* were rarely found and are thus considered to be an indicator of possible microbial contamination of the fresh produce, in particular, fecal contamination^33^,^34^,^35^. These results were confirmed by both IS-Pro bacterial profiles and culturing (Fig. 2b-2).

Hence, for our biosensing experiments the process water were spiked with different concentrations of *E. coli* K-12 bacteria. The presence of *E. coli* in the spiked process water was confirmed by both IS-Pro analysis and culturing (see Fig. 2a-3 and 2b-3, respectively). The IS-Pro bacterial profile of the spiked process water contains characteristic *E. coli* peaks, marked with arrows for clarity, in addition to the typical characteristic profile of this water and in agreement to the molecular fingerprinting of a neat *E. coli* suspension (Fig. 2a-1). Moreover, the culturing results using *E. coli* specific medium (Fig. 2b) correspond to the IS-Pro analysis (Fig. 2a).

Recently, we have demonstrated the potential of PSi-based biosensors to detect *E. coli* bacteria in laboratory suspensions at relatively low bacterial concentrations (in the range of 10^3^–10^5^ cells/mL) within minutes^25^,^26^. Herein, we attempt to extend the capability of these biosensors to perform in complex samples at point-of-care to allow rapid assessment of food safety during early processing^42^. In order to meet the challenges of the complex samples, the biosensors were optimized in terms of their surface chemistry. Our previously-reported biosensors^26^, in which we used NHS ester reaction to immobilize the amine-terminated IgG to the PSiO_2_ surface, were not stable in process water (data not shown). Therefore, the a streptavidin/biotin-mediated conjugation of antibodies was employed and a final capping step with ethanolamine, for minimizing non-specific binding.

The nanostructured PSiO_2_ was functionalized with *E. coli*-specific antibodies through silanization chemistry followed by biotin-SA coupling (see Fig. 3b). The attachment of the antibodies to the PSi surface was confirmed by fluorescent labeling experiments, followed by observation of the biosensors under a fluorescence microscope. Figure S1 (Supplementary Information) summarizes the results of these experiments. The activity and specificity of the *E. coli* antibodies (produced in rabbit) conjugated to the PSi were confirmed by binding of fluorescently tagged anti-rabbit IgG. On the other hand, exposure of the biosensors to fluorescently tagged anti-mouse-IgG showed a negligible fluorescence signal. The latter is ascribed to minor non-specific binding of the anti-mouse IgG to the biosensor surface.

The biosensing concept, illustrated in Fig. 4, is based on monitoring changes in the light reflected from the IgG-modified PSiO_2_ in real time. Exposure of the biosensors to process water spiked with culture-grown *E. coli* (in addition to their natural high microbial load) resulted in specific *E. coli* capture onto the IgG-modified nanostructure via antibody-antigen interactions. Cells capture is detectable, as it induced a robust decrease in the intensity of the reflected light (Fig. 4c) and was further confirmed by optical micrographs of the biosensor surface (Fig. 4d).

For biosensing experiments, the process water samples were spiked with different *E. coli* concentrations ranging from 10^3^ to 10^5^ cells/mL. Figure 5 presents the results of a representative biosensing experiment in real time in terms of changes in the FFT peak intensity vs. time. Upon introduction of the process water (containing 10^4^ cells/mL *E. coli*), a significant intensity decrease of 5% was obtained, attributed to binding of the target cells to the IgG-modified surface. As we demonstrated previously^25^,^26^,^27^, bacteria are excluded from penetrating into the nanostructure due to their size (typical dimensions of 0.8–2 μm^43^) and are therefore captured onto the surface. This in turn induces light scattering and results in a significant decrease in the intensity of the reflected light. It is important to note the stability of the intensity signal during the rinsing step (see Fig. 5a), indicating that the captured cells were tightly bound to the antibody under these conditions. When original process water (no *E. coli*) were introduced to the biosensor, the intensity is observed return to its original baseline value after removal of the process water and rinsing with saline (see Fig. 5b), suggesting that no capture events have occurred.

To validate the results of these experiments, all biosensors were studied by both light and electron microscopy. Observation of the biosensors immediately after the optical experiments by both light microscopy and HRSEM confirms the presence of immobilized bacteria cells onto the surface (Fig. 5c), while no cells were observed in the control experiments with the original water (Fig. 5d). These results support our approach that direct cell capture of bacteria onto the PSiO_2_ surface via antibody-antigen interactions can be observed by monitoring changes in the intensity of the reflectivity spectrum. Next, in order to confirm the specificity of the biosensors towards *E. coli* bacteria, the cells were recovered from the biosensors and were analyzed by real-time PCR. The results are summarized in Table 1, revealing that the DNA extracted and isolated from the biosensors indeed fits to that of *E. coli*. Moreover, the PCR quantification shows that the number of captured cells on the biosensors’ surfaces correlates to their concentration in the process water. To best of our knowledge, this is the first time that cells were recovered from a biosensor and thoroughly analyzed by real-time PCR to confirm the sensor performance in terms of its selectivity.

In Fig. 6, the averaged optical response of the biosensors upon introduction of process water spiked with different concentrations of *E. coli* is presented. The intensity signals are proportional to the target bacteria concentration. Thus, exposure of the biosensors to increasing *E. coli* concentrations results in a larger change in signal, i.e., a larger decrease in intensity can be observed (see Fig. 6a). The biosensors produced reproducible signals and the values obtained for each studied concentration are statistically significant (t-test, *p* < 0.05). Moreover, an excellent linear correlation (R^2^ = 0.9883) is demonstrated to the *E. coli* bacterial concentration in the range of 10^3^ to 10^5^ cells/mL (see Fig. 6b) and the measured limit of detection (LoD) is 10^3^ cells/mL. It should be noted that to the best of our knowledge, this is the first report that demonstrates sensitive target bacteria detection of 10^3^ cells/mL in real food industry water (containing high load of unknown wild type bacterial species, plant organisms and dirt), without any pre-enrichment or prior processing steps and a total assay time of 45 minutes. Moreover, although the biosensors capability still does not meet the microbial standards for drinking water in the Netherlands (0 CFU/100 mL for total coliforms and fecal coliforms)^44^, their ability to selectively detect and quantify the target bacteria in samples containing mixed bacterial populations at high bacterial loads (5 × 10^7^ cells/mL) is noteworthy. In particular, as the majority of literature-reported work on biosensors presents their performance in simplified media e.g., buffer with target analyte, avoiding real environmental samples, in which consideration aspects such as sample pretreatment, matrix effects are crucial^45^,^46^.

In conclusion, this work demonstrates the application of new label-free optical immunosensors to real-world complex samples and discusses challenges associated with the analysis of such samples. These samples contained a high microbial load of various non-target microorganisms as well as soil particles and plant cell debris. The biosensors were capable of selectively identifying and quantifying the target cells, while the target cell concentration was orders of magnitude lower than that of other bacterial species. We achieved one order of magnitude improvement in the sensitivity in comparison with our previous studies^26^,^31^ in which neat bacterial suspensions were studied. In terms of the detection limit of the biosensor, the biosensor demonstrates a measured LoD of 10^3^ cells/mL in real food industry water without any pre-enrichment or prior processing steps and a total assay time of 45 minutes. Currently we are exploring several approaches to enhance the sensitivity of the biosensor, including optimization of antibody concentration and orientation, via different coupling chemistries and incorporation of other capture probes, e.g., antibody fragments and aptamers. Moreover, we are developing strategies for integration of our biosensors with lab-on-a-chip approaches to facilitate their performance and achieve higher sensitivity together with rapid speed for bacteria detection. The presented biosensing platform is not only portable but is designed to remotely detect bacteria in the factory or field and indeed several preliminary experiments were successfully carried at the food factory site. Thus, our system has the potential to detect contaminations in product lines and expedite the decision-making process, plant sanitation, and product processing. Such technologies can facilitate the reuse of water, as they have the potential to be used as a screening tool to rule out pathogens in product lines and decrease the risk of cross contamination and spread of pathogens.

## Methods

### Preparation of Porous SiO_2_

Single-side polished and heavily doped p-type Si wafers (0.001 Ω-cm resistivity, 〈100〉 oriented, B-doped from Sil’tronix Silicon Technologies, France) were electrochemically etched in a 3:1 (v/v) solution of aqueous HF (48%, Merck, Germany) and ethanol (99.9%, Merck, Germany) for 30 s at a constant current density of 385 mA/cm^2^. Si wafers with an exposed area of 1.33 cm^2^ were contacted on the backside with a strip of aluminum foil and mounted in a custom-made Teflon etching cell; a platinum ring was used as the counter-electrode. After etching, the surface of the wafer was thoroughly rinsed with ethanol and dried under a stream of dry nitrogen gas. Subsequently, the freshly-etched PSi samples were thermally oxidized in a tube furnace (Thermolyne) at 800 °C for 1 h in ambient air.

Note: All the materials were purchased from Sigma Aldrich Chemicals unless otherwise mentioned.

### Scanning Electron Microscopy

High-resolution scanning electron microscopy (HRSEM) studies of the PSiO_2_ biosensors immediately after the biosensing experiments were carried out using a Carl Zeiss Ultra Plus HRSEM at an accelerating voltage of 1 keV. The biosensors were fixed using a glutaraldehyde solution (2% in 0.1 M PBS) followed by dehydration through an ethanol series (10% to absolute). Phosphate-buffered saline (PBS) at pH = 7.4 was prepared by dissolving 50 mM Na_2_HPO_4_, 17 mM NaH_2_PO_4_, and 68 mM NaCl in Milli-Q water (18.2 mΩ-cm). Subsequently, the biosensors were sputtered with carbon.

### Measurement of Interferometric Reflectance Spectra

Interferometric reflectance spectra of the samples were collected using a CCD spectrometer (Ocean Optics, USB4000) fitted with a microscope objective lens coupled to a bifurcated fiber optic cable. A tungsten light source was focused onto the center of the sample surface with a spot size approx. 1–2 mm^2^. Reflectivity data were recorded in the wavelength range of 400–1000 nm with a spectral acquisition time of 100 ms. Both illumination of the surface and detection of the reflected light were performed along an axis coincident with the surface normal. All optical experiments were conducted in a fixed cell in order to assure that the sample reflectivity is measured at the same spot during all the measurements. Spectra were collected in real time and analyzed by applying fast Fourier transform (FFT).

### Biofunctionalization of PSiO_2_ Scaffolds

A PSiO_2_ sample was incubated with aqueous solution of 42 mM 3-aminopropyl(triethoxyl)silane (APTES) and 56 mM diisopropylethylamine (DIEA) for 30 min. After the removal of the solution, the surface was rinsed with purified water and ethanol for 10 min each and dried under a nitrogen stream. The resulting amine-terminated nanostructure was functionalized with 2% (v/v) glutaric di-aldehyde (25 wt%) (GluAld) aqueous solution for 30 min. Subsequently, the surface was washed with purified water and dried. A solution of sodium cyanoborohydride (10 μL) in HEPES (4-(2-hydroxyethyl)-1-piperazineethanesulfonic acid) buffer (1 mL) was pipetted onto the GluAld-modified PSiO_2_ and incubated for 30 min in order to stabilize the Schiff base, which is formed during reaction of the aldehyde groups with the amine groups. HEPES buffer solution at pH = 8 was prepared by dissolving 0.1 M HEPES and 0.1 M NaCl in Milli-Q water. Next, the surface was thoroughly rinsed with HEPES buffer and incubated in streptavidin (SA, from Jackson ImmunoResearch Labs Inc., USA) solution (100 μg/mL in PBS) for 1 h. Then, the reducing step with sodium cyanoborohydride was repeated in order to stabilize the Schiff base formed during SA fixation. In order to minimize non-specific binding, unreacted aldehyde groups on the PSiO_2_ were terminated using ethanolamine (3 M in BBS, incubation of 30 min)^38^,^47^. Borate buffered Saline (BBS) solution (0.15 M) at pH = 9 was prepared by dissolving 1 M boric acid and 1 M sodium tetra borate 10-hydrate in Milli-Q water. IgG-modified PSiO_2_ was achieved by incubation of the SA-modified surface with 100 μg/mL biotinylated *E. coli* rabbit antibody solution (from RayBiotech Inc., USA) for 1 h. The resulting biosensors were kept at 4 °C until used.

### Fluorescent Labeling and Fluorescence Microscopy

Biosensors (IgG-modified PSiO_2_) were incubated with fluorescein (FITC)-conjugated anti-rabbit IgG (1:50 v/v dilution of manufacturer’s stock solution, from Jackson ImmunoResearch Labs Inc., USA) for 40 min. Biosensors were also incubated with fluorescein (FITC)-conjugated anti-mouse IgG as a control (1:50 v/v dilution of manufacturer’s stock solution, from Jackson ImmunoResearch Labs Inc., USA). Following conjugation, the samples were observed under a fluorescence microscope (Zeiss Axio) and images were taken using an AxioCamMRc camera. A constant exposure time of 4 s was used for all measurements. Data were analyzed by AxioVision and Imaris Bitplane scientific software.

### Process water samples handling

Process water samples from a Dutch fresh-cut produce company were collected from different washing lines. The water samples were treated with 0.05 mg/mL tetracycline hydrochloride and kept at 4 °C, in order to prevent the growth of the microorganisms^48^ and allow the usage of a single batch of water over time. The effects of antibiotics and storage were studied by comparing fresh water (no antibiotics) with 1-month stored water. Water samples were cultured and similar bacterial loads were measured. For biosensing experiments, the process water were spiked with different concentrations of *E. coli* K-12 (generously supplied by Prof. Sima Yaron, Technion, Israel).

### Bacteria Culture

The total bacterial load of the water samples was determined by plating 1 mL of dilution series of the process water in plate count agar (PCA) medium (Becton, Dickinson and Company (BD), USA) using the pour plate technique, and incubating at 30 °C for 72 h.

*E. coli* count was determined by plating 100 μL of process water on Eosin Methylene Blue Agar (EMB Agar) medium (Becton, Dickinson and Company (BD), USA), which is a slightly selective and differential medium for the isolation and differentiation of Gram-negative enteric bacilli from clinical specimens. Colonies of *E. coli* on this specific medium appear as blue-black, with a green metallic sheen. Process water samples were incubated on an EMB agar medium at 37 °C for 24 h.

For biosensing experiments, *E. coli* K-12 were overnight grown in Luria Broth (LB) medium at 37 °C with shaking. LB medium was prepared by dissolving 5 g NaCl, 5 g yeast extract and 10 g tryptone (Becton, Dickinson and Company (BD), USA) in 1 L deionized water.

Bacterial concentration was monitored photometrically by reading the optical density (OD) at a wavelength of 600 nm. The number of cells is directly proportional to the OD_600_ measurements (1 OD_600_ = 3 × 10^8^ cells/mL) and bacteria concentration is calculated from the obtained OD_600_ values. The correlation between bacteria concentration and OD_600_ measurement was determined empirically.

### DNA isolation

Process water (500 mL) were filtered through a 0.22 μm filter. Filters and biosensors were incubated in 600 μL lysis buffer (EasyMaglysis buffer, Biomerieux). DNA was purified from 500 μL lysate using a Chemagen DNA isolation robot (PerkinElmer) and eluted into 100 μL of 10 mM Tris/HCl (pH = 8.0) and 1 mM ethylenediaminetetraacetic acid (EDTA).

### IS-Pro analysis

Bacterial profiles of process water samples were determined using IS-Pro technology. IS-Pro involves bacterial species differentiation by the length of the 16S–23 S rRNA genes interspace region with taxonomic classification by phylum-specific fluorescent labeling of PCR primers. The IS-pro procedure consists of two multiplex PCRs: a first PCR for the phyla *Firmicutes, Bacteroidetes, Actinobacteria, Fusobacteria* and *Verrucomicrobia* and a second PCR for the phylum *Proteobacteria*^32^,^49^. 10 μL DNA was used in each PCR and the PCR products were separated using an ABI Prism 3130XL Genetic Analyzer. The results are presented as color-labeled peak profiles. All data visualizations were performed with the Spotfire software package (TIBCO, Palo Alto, CA, USA).

### Real-time PCR

Total bacterial loads and presence of *E. coli* bacteria on the biosensors’ surfaces (after sensing experiments) were measured by real-time PCR using primers targeting conserved regions in the 16S rRNA gene^50^. Quantification was performed using a standard consisting of the *E. coli* amplicon cloned into plasmid pGEM-T Easy (from Promega, USA). The amount of plasmid was calculated from A260 measurements. In this way, total copies of 16 S rRNA gene can be calculated. To transform this number into bacterial loads we used a mean of 4 16 S rRNA gene copies per bacterial cell. To measure specific *E. coli* loads we used an established *E. coli*-specific real-time PCR assay^51^.

### Bacteria Biosensing

Biosensors (IgG-modified PSiO_2_) were incubated with process water spiked with *E. coli* K-12, with concentrations ranging from 10^3^ to 10^5^ cells/mL, for 30 min. The original process water (no *E. coli*) were used as a control. In a typical experiment, the biosensor was fixed in a custom-made flow cell and a saline solution was introduced for 15 min for establishing optical baseline readout. Subsequently, 10 mL of process water was introduced and allowed to incubate for 15 min. Next, the cell was flushed for 15–30 min with saline (to remove non-target microorganism and dirt that may reside on the biosensor surface) until a constant optical signal was attained. Optical measurements were recorded throughout the experiment. The optical signal, i.e., FFT intensity change, is expressed in this work as percentage and was calculated using the following equation:


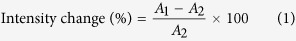


where *A*_1_ is the averaged intensity value collected from the biosensor during baseline establishment and *A*_*2*_ is the averaged intensity attained after exposure of the biosensor to the process water sample and subsequent washing. The detection limits were calculated from the linear correlation plot (see Fig. 6) using 3*S*_*e*_*/m*, where *S*_*e*_ is the standard of error and m is the slope.

It should be also noted that the effect of antibiotics addition to the sampled water was studied. Biosensing experiments were carried out on freshly-sampled water (with and without the tetracycline treatment), and the antibiotic addition did not interfere with the biosensing results.

### Statistical analysis

Statistical analysis is performed using a Student’s t-test with a minimum confidence level of 0.05 for statistical significance and assuming unequal sample sizes and unequal variance. All values are reported as the mean and standard deviation of the mean.

## Additional Information

**How to cite this article**: Massad-Ivanir, N. *et al*. Porous Silicon-Based Biosensors: Towards Real-Time Optical Detection of Target Bacteria in the Food Industry. *Sci. Rep.* **6**, 38099; doi: 10.1038/srep38099 (2016).

**Publisher's note:** Springer Nature remains neutral with regard to jurisdictional claims in published maps and institutional affiliations.

## Supplementary Material

Supplementary Information

## Figures and Tables

**Table 1:** Results of real-time PCR analysis of biosensor surfaces following biosensing experiments.

*E. coli* concentration (cells/mL)	Intensity decrease (%)	*E. co*li (Cells per biosensor)
10^3^	3	6650
10^5^	7	27230
No *E. coli* (control)	0	6
