# Supplementary material for: Porous Silicon-Based Biosensors: Towards Real-Time Optical Detection of Target Bacteria in the Food Industry
Source: Sci Rep. 2016 Nov 30;6:38099. doi: 10.1038/srep38099 (PMC5128872; doi:10.1038/srep38099)
Supplement: Supplementary Information [file srep38099-s1.doc]

**Supplementary Information**

**Porous Silicon-Based Biosensors: Towards Real-Time Optical Detection of Target Bacteria in the Food Industry**

Naama Massad-Ivanir^1§^, Giorgi Shtenberg^2§^, Nitzan Raz^1^, Christel Gazenbeek^3^, Dries Budding^4^, Martine P. Bos^3^, and Ester Segal^1,5^*

^1^Department of Biotechnology and Food Engineering, Technion – Israel Institute of Technology, Haifa 32000, Israel.

^2^The Interdepartmental Program of Biotechnology, Technion – Israel Institute of Technology, Haifa 32000, Israel.

^3^Microbiome Ltd, Amsterdam, the Netherlands.

^4^Department of Medical Microbiology, VU medical Center, Amsterdam, the Netherlands.

^5^The Russell Berrie Nanotechnology Institute, Technion – Israel Institute of Technology,

^§^Equal contribution

*Correspondence should be addressed to E.S. ([esegal@tx.technion.ac.il](mailto:esegal@tx.technion.ac.il))

**Figure S1:** Results of ﬂuorescence experiments to conﬁrm the modiﬁcation of the PSiO_2_ surfaces with antibodies. The binding of the *E. coli* antibodies to the PSiO_2_ nanostructure is conﬁrmed by exposing the biosensors to a ﬂuorescently tagged anti-rabbit IgG followed by observation of the films under a ﬂuorescence microscope. The fluorescence is quantified by image analysis (performed by Imaris Bitplane scientiﬁc software). FITC-anti-rabbit IgG bind only to biotinylated rabbit IgG species (complete biofunctionalization), while no binding of unspeciﬁc ﬂuorescent antigen (FITC-anti-mouse IgG) is observed, control 1. In another control experiment (control 2), the IgG conjugation step is omitted, and no ﬂuorescence is observed, indicating the lack of unspeciﬁc attachment between the anti-rabbit IgG and the SA-modified PSiO_2_ surface.
